# Bilateral Post-Traumatic Carotid-Cavernous Fistula: A Case Report

**DOI:** 10.3390/reports9030249

**Published:** 2026-08-01

**Authors:** Ondrej Placek, Tomas Krejci, Radim Lipina, Vaclav Prochazka

**Affiliations:** 1 Department of Neurosurgery, University Hospital Ostrava, 708 00 Ostrava, Czech Republic; ondrej.placek@fno.cz (O.P.); radim.lipina@fno.cz (R.L.); 2 Department of Radiology, University Hospital Ostrava, 708 00 Ostrava, Czech Republic; vaclav.prochazka@fno.cz

**Keywords:** carotid-cavernous fistula, flow-diverter, embolization

## Abstract

**Background and Clinical Significance:** Carotid-cavernous fistulas (CCFs) are pathological communications between the carotid artery and the cavernous sinus, most commonly traumatic when direct and high-flow (Barrow type A). Bilateral traumatic CCFs are rare, occurring in approximately 1–2% of cases. **Case Presentation:** We report a 45-year-old male with polytrauma after a road traffic accident who presented with right-sided chemosis, pulsatile exophthalmos, and ocular bruit. Digital subtraction angiography revealed bilateral direct CCFs. The right fistula was treated with transarterial coil embolization combined with flow-diverter stent placement in the intracavernous internal carotid artery. Subsequent imaging demonstrated left-hemispheric ischemia while the contralateral high-flow CCF remained untreated; the underlying mechanism was considered potentially hemodynamic or thromboembolic. The left fistula was managed using the same technique. Final angiography confirmed complete bilateral occlusion. Despite transient postoperative epistaxis, the patient showed neurological improvement. **Conclusions:** This case highlights the effectiveness of combined flow diversion and coiling in managing rare bilateral traumatic CCFs.

## 1. Introduction and Clinical Significance

A carotid-cavernous fistula (CCF) is an abnormal arteriovenous communication between the carotid arterial system and the cavernous sinus. According to the Barrow classification, CCFs are divided into direct (Type A) and indirect (Types B–D) lesions. Direct CCFs are high-flow fistulas, most commonly resulting from craniofacial trauma, whereas indirect fistulas are typically low-flow dural shunts [1,2,3].

Traumatic direct CCFs account for the majority of cases and usually arise after skull base fractures involving the cavernous segment of the internal carotid artery (ICA) [4,5]. The resulting arteriovenous shunt leads to venous hypertension within the cavernous sinus and orbital venous system, producing characteristic clinical signs such as pulsatile exophthalmus, chemosis, orbital bruit, cranial nerve palsies, and visual impairment [1,4]. In severe cases, cortical venous reflux may lead to intracranial hemorrhage [6].

Digital subtraction angiography (DSA) remains the gold standard for diagnosis and treatment planning, as it provides detailed hemodynamic and anatomic characterization of the fistula. While CT and MR angiography may be suggestive, they are insufficient for definitive evaluation [1,2].

Endovascular therapy is the first-line treatment for CCFs, aiming to eliminate the fistula while preserving ICA patency. Available techniques include transarterial or transvenous embolization using coils or liquid embolics, balloon occlusion, covered stents, and flow-diverter stents. Flow diversion has emerged as a reconstructive option that promotes gradual fistula closure through vessel wall remodeling and is often combined with coiling in high-flow lesions to improve occlusion rates [1,2,7].

Bilateral traumatic CCFs are extremely rare, reported in only 1–2% of cases, and optimal management is not well established. This rarity limits available evidence to case reports and small series, underscoring the relevance of individual case contributions to the literature [8,9,10].

## 2. Case Presentation

**Figure 1:**
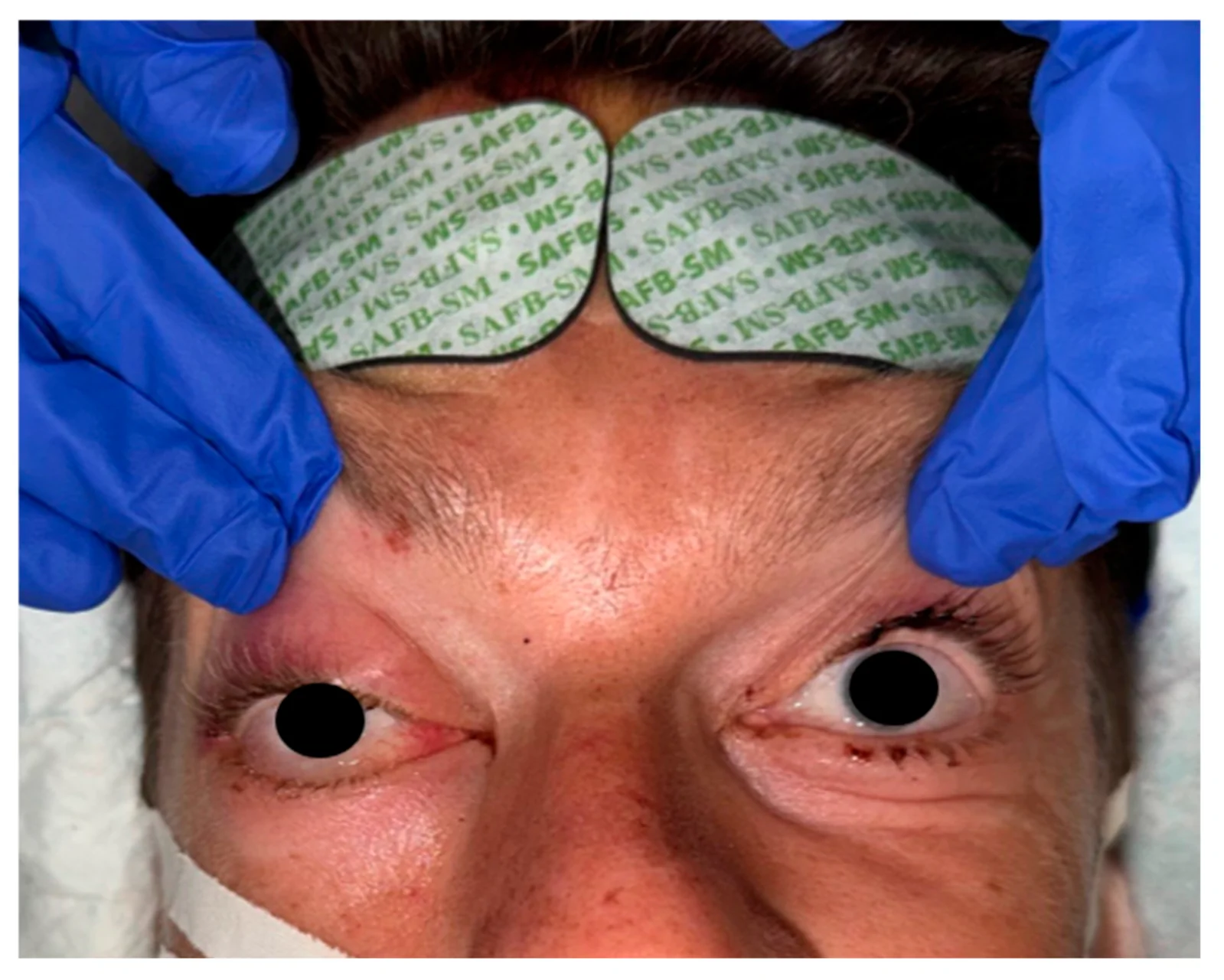
The patient, unconscious, presenting with conjunctival chemosis and right-sided exophthalmus.

A 45-year-old male with multiple severe traumatic injuries was admitted to the Trauma Centre of the University Hospital Ostrava following a high-energy road traffic accident, initially in an unconscious state. The patient required immediate airway management with endotracheal intubation and hemodynamic stabilization according to standard trauma protocols. On primary ophthalmological and neurological examination, marked right-sided conjunctival chemosis, proptosis (exophthalmos), and an audible orbital bruit were observed (Figure 1), raising strong suspicion of a vascular injury involving the cavernous sinus region. Ophthalmological assessment confirmed significantly elevated intraocular pressure on the right side, while the left eye remained clinically asymptomatic at presentation.

**Figure 2:**
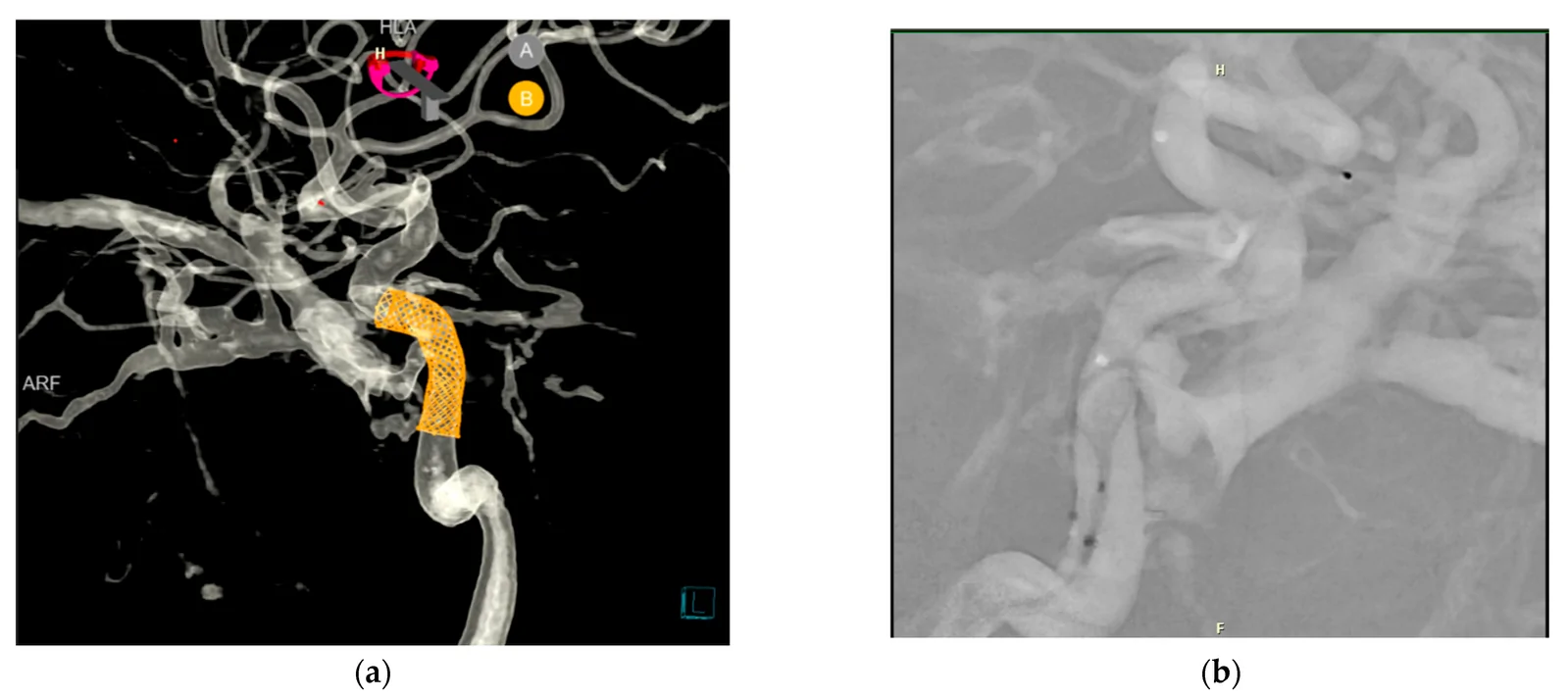
(**a**) Initial DSA demonstrating a right CCF, for which virtual flow-diversion modelling was conducted for its eventual occlusion, and typical dilation of the superior ophthalmic vein. (**b**) Flow diversion deployed at the site of the right fistula.

Given the clinical suspicion of carotid-cavernous fistula (CCF), emergent digital subtraction angiography (DSA) was performed. Imaging demonstrated direct bilateral carotid-cavernous fistulas with high-flow shunting from the intracavernous segments of the internal carotid arteries into the cavernous sinuses (Figure 2a), confirming severe traumatic vascular injury. Following diagnostic angiography, dual antiplatelet therapy consisting of acetylsalicylic acid and clopidogrel was initiated, and adequate platelet inhibition was confirmed prior to endovascular reconstruction. Dual antiplatelet therapy was continued for 6 weeks, followed by long-term acetylsalicylic acid monotherapy. Subsequently, staged endovascular treatment was performed, beginning with the right-sided lesion. Through a combined transarterial and trans-fistulous approach, six Optima detachable coils were deployed into the cavernous sinus via the fistulous tract to induce flow stasis and promote thrombosis. In addition, a P64 flow-diverter stent was implanted (Figure 2b). Post-procedurally, rapid clinical improvement was observed, including regression of right-sided intraocular pressure and reduction in orbital congestion.

**Figure 3:**
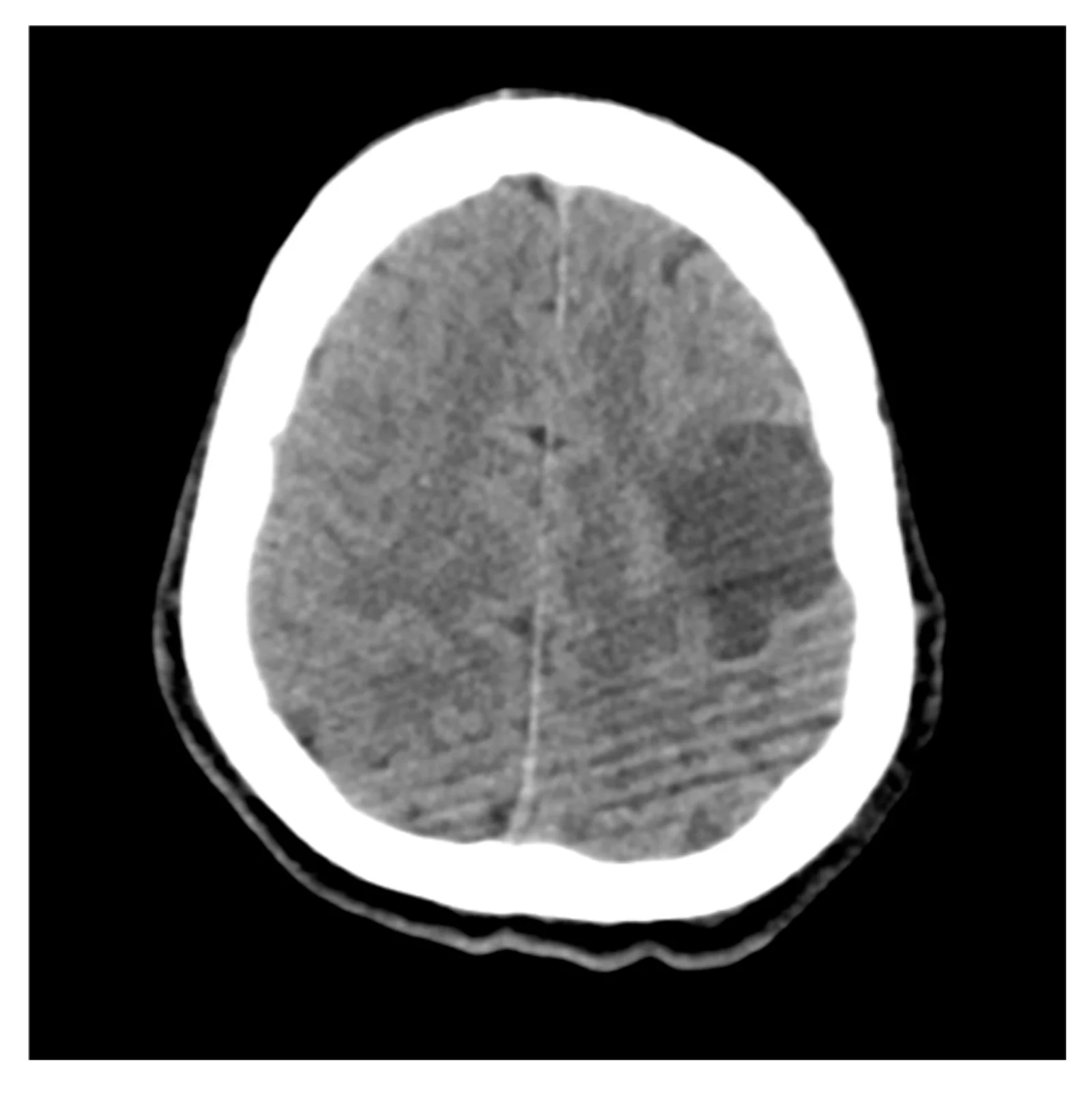
Follow-up CT imaging demonstrating new left frontoparietal ischemia.

**Figure 4:**
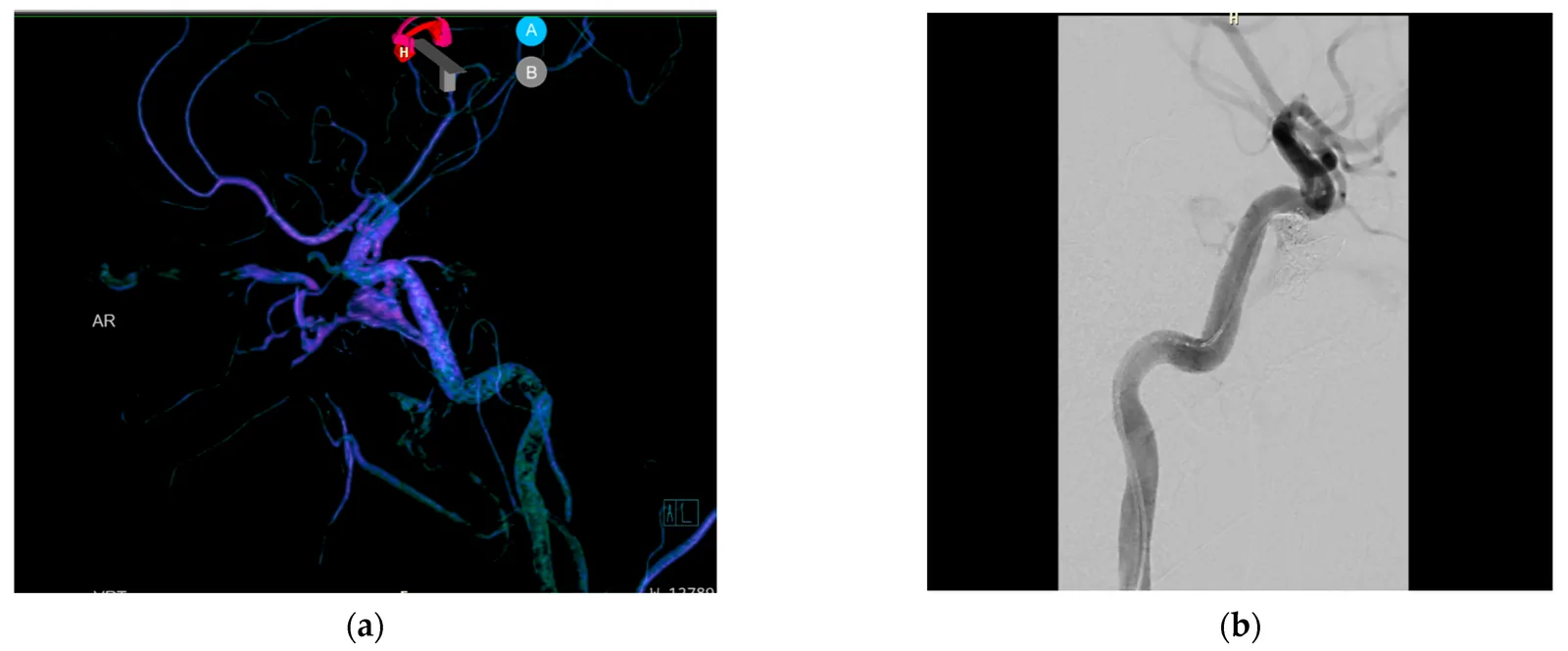
(**a**) DSA demonstrating the persistent untreated left-sided CCF. (**b**) Introduction of flow-diverter stent in the left-sided fistula with confirmed occlusion.

On the following day, routine follow-up computed tomography (CT) of the brain revealed a new ischemic lesion in the left cerebral hemisphere (Figure 3), raising suspicion of either thromboembolic complication or hemodynamic compromise in the setting of bilateral vascular intervention and antiplatelet therapy. Repeat DSA confirmed persistence of a high-flow left-sided carotid-cavernous fistula (Figure 4a), which was subsequently treated using the same endovascular strategy. Three Optima detachable coils were deployed into the cavernous sinus, followed by implantation of a P64 flow-diverter stent (Figure 4b). Final angiography demonstrated complete occlusion of both fistulas with preserved patency of the parent arteries.

In the post-interventional period, the patient developed significant bilateral epistaxis, more pronounced on the right side, secondary to venous hypertension and mucosal vessel congestion in the setting of cavernous sinus outflow alteration. Initial management with nasal tamponades was insufficient to control the bleeding, necessitating evacuation of clots from the oral cavity and temporary tracheostomy to secure the airway and prevent aspiration. Hemostasis was achieved after repositioning and replacement of nasal tamponades, with no recurrence of epistaxis after 3 days, allowing their removal. At the time of transfer to the referring facility, the patient was hemodynamically stable, fully conscious, able to obey simple commands, with regressing right-sided hemiparesis and the ability to maintain a standing position for short intervals. His overall neurological and functional status remained influenced by the severity of the initial polytrauma and associated complications.

## 3. Discussion

Bilateral traumatic CCFs, as in the presented case, are rare, being observed in 1–2% of CCF cases in the literature [11]. Bilateral fistulas require timely and comprehensive intervention. The mechanism underlying the postoperative left hemispheric ischemia remains uncertain. Several potential mechanisms may have contributed to the ischemic event, including a steal phenomenon related to the persistent high-flow contralateral carotid-cavernous fistula [1,3], thromboembolic events related to the initial endovascular procedure, transient hemodynamic alterations following flow-diverter deployment, or other factors associated with the patient’s critical condition and dual antiplatelet therapy. However, none of these mechanisms can be confirmed based on the available data, and the exact etiology of the infarction should therefore be regarded as speculative. Spontaneous bilateral CCFs are also rarely observed [8].

Although bilateral CCFs were identified at initial angiography, treatment was performed in a staged manner to reduce procedural complexity in this critically injured patient. The occurrence of cerebral ischemia before treatment of the second fistula highlights the potential risk of hemodynamic or thromboembolic complications associated with an untreated contralateral lesion. However, based on this single case, it cannot be determined whether the staged treatment contributed to the ischemic event, and no conclusion can be drawn regarding the superiority of simultaneous or immediate bilateral treatment. Nevertheless, early treatment of both fistulas should be considered whenever clinically feasible, taking into account the patient’s clinical condition and procedural risk.

Epistaxis following endovascular occlusion is considered a rare but clinically significant complication, with an incidence of <1% in larger datasets, although these are often based on individual case reports. Some authors associate this complication with residual drainage, iatrogenic trauma, or recanalization of the fistula with diversion of venous drainage towards areas communicating with the nasal cavity leading to elevated pressure in the mucosa and bleeding [4,5]. Control DSA is recommended in cases of repeated tamponade failure.

Experience with flow diversion as a treatment modality in the management of CCFs has increased in recent years. Hüseyinoglu et al. [7] summarized 27 cases of direct CCFs treated with flow diversion, with complete occlusion achieved in all patients. Similarly, Stamatopoulos et al. [2], in a study of 38 patients, reported clinical improvement in 92.1% of patients and long-term fistula occlusion in all cases. Our experience is consistent with these findings, demonstrating the potential efficacy of flow diversion combined with coiling. However, the main disadvantage of flow diversion is the requirement for dual antiplatelet therapy, which may increase the risk of bleeding complications, particularly in severely injured trauma patients, and should therefore be carefully considered when selecting the optimal treatment strategy.

Additional methods of CCF closure were described by Li et al., who, in their case series, reported lesion obliteration using coils, detachable balloons, and Onyx liquid embolic agent [6]. In contrast, Camara et al. managed closure of CCF using coil embolization alone [10].

## 4. Conclusions

The case presented here demonstrates that, even in critically injured patients, bilateral CCFs can be managed successfully with endovascular therapy combining flow diversion with coiling. Bilateral CCF occlusion resulted in angiographic and clinical regression of symptoms, with only mild hemiparesis persisting. A key factor is the early diagnosis and treatment of both lesions. Both the literature and this case report highlight high success rates with flow diversion in combination with coiling. This case underscores the importance of close monitoring because of the possibility of early complications, even following apparently successful interventions. Long-term fistula occlusion and improvement in neurological condition have been noted in the majority of studies.

## Data Availability

Data supporting the findings of this case report are available from the corresponding author upon reasonable request. Data are not publicly available due to patient privacy considerations.
